# Intentional Binding Effects in the Experience of Noticing the Regularity of a Perceptual-Motor Task

**DOI:** 10.3390/brainsci10090659

**Published:** 2020-09-22

**Authors:** Kazuki Hayashida, Yuki Nishi, Akihiro Masuike, Shu Morioka

**Affiliations:** 1 Department of Neurorehabilitation, Graduate School of Health Sciences, Kio University, 4-2-2 Umaminaka, Koryo, Kitakatsuragi-gun, Nara 635-0832, Japan; kazuki_aka_linda@yahoo.co.jp (K.H.); index2424@gmail.com (Y.N.); 2 Department of Rehabilitation, Fujiikai Rehabilitation Hospital, 17-6 Yayoi-cho, Higashiosaka-city, Osaka 579-8026, Japan; 3 Department of Rehabilitation, Higashiosaka Yamaji Hospital, 1-7-5 Inaba, Higashiosaka-city, Osaka 578-0925, Japan; b4107270@kio.ac.jp; 4 Neurorehabilitation Research Center, Kio University, 4-2-2 Umaminaka, Koryo, Kitakatsuragi-gun, Nara 635-0832, Japan

**Keywords:** intentional binding, experience of noticing, motor performance

## Abstract

Noticing the regularity of the task is necessary to enhance motor performance. The experience of noticing further motivates improvement in motor performance. Motor control is explained by a comparator model that modifies the motor command to reduce discrepancies between sensory predictions and actual outcomes. A similar model could apply to sense of agency (SoA). SoA refers to the sensation of controlling one’s own actions and, through them, the outcomes in the external world. SoA may also be enhanced by the experience of noticing errors. We recently reported gradual enhancement of SoA in participants with high perceptual-motor performance. However, what component of the motor task changed the SoA is unclear. In this study, we aimed to investigate the influence over time of the experience of noticing during a motor task on SoA. Participants performed an implicit regularity perceptual-motor task and an intentional binding task (a method that can quantitatively measure SoA) simultaneously. We separated participants into groups after the experiment based on noticing or not noticing the regularity. SoA was gradually enhanced in the noticing group, compared with that of the non-noticing group. The results suggest that the experience of noticing may enhance SoA during perceptual-motor tasks.

## 1. Introduction

In many areas of human life, the experience and repetition of many situations is required to improve motor performance. In the repeating process, noticing the stimulus feature or regularity required to achieve the purposes of a task improves motor performance. The experience of noticing, which is caused by predictions about the outcome and errors in performance, induces positive emotions and further motivates one to improve motor performance (i.e., reinforcement learning occurs) [1,2,3]. Motor control or motor learning is explained by a comparator model that modifies the motor command to reduce the discrepancy between sensory prediction and the actual outcome [4,5]. A similar model is considered for application to a sense of agency (SoA) in embodiment systems [6,7,8,9]. Embodiment is distinguished into sense of ownership (SoO) and SoA. SoO refers to the perception of one’s own body, whereas SoA refers to the sensation of controlling one’s own actions and, through them, outcomes in the external world [10]. Therefore, it has been suggested that SoA is directly related to motor control. If the discrepancy between sensory prediction and outcome is small, SoA will arise. Meanwhile, if the discrepancy is large, SoA will not arise [11,12,13,14,15]. That is, SoA may also be enhanced by emotions (e.g., a eureka experience [16]) caused by the experience of noticing the relationship between predictions and errors, as with motor learning.

Motor control and SoA, in terms of the theory of prediction and error, may be closely related. The comparator model, however, cannot explain some aspects of the experience of agency. For example, SoA increases when there is a clear behavioral goal [17]. Goals and rewards are highly associated, and it has been suggested that a goal-directed action affects agency [18]. Previous studies have used an experimental paradigm in which the actions of the participant resulted in an audible tone, which is associated with rewards. In the study, the intentional binding (IB) effect (a method that can quantitatively measure SoA [19,20,21,22]) was reduced in the penalty trials compared with that in the neutral or reward trials [23]. These findings indicate the influence of rewards on the effects of IB. Dopaminergic neurons function in the reward system and are heavily involved in reinforcement learning. Interestingly, dopaminergic medication, such as levodopa, increases the IB effect in patients with Parkinson’s disease, a condition characterized by dopamine depletion [24]. Therefore, SoA may be reinforced in an ambitious motor task with goals and rewards.

We recently investigated this relationship using an IB that included motor tasks with goals and rewards [25]. In our previous study, participants stopped a moving object at a certain speed by a keypress when it reached the target on the display screen. This task also included an additional exercise: the IB task. After pressing the key, a sound was presented after a randomly chosen time interval of 200, 500, or 700 ms, respectively, and the participants had to estimate the time intervals between their actions (pressing the key) and the outcomes (tone). The perceived shorter time interval is used as an index of a greater IB effect (i.e., enhancement of SoA). We showed a gradual enhancement of the IB effect in participants with high perceptual-motor adaptation effects (i.e., a strong goal-oriented action to achieve the task).

The results supported the hypothesis that perceptual-motor adaptation and SoA are adapted to the comparator model. However, what component of the adaptation task changed the SoA is unknown. In the previous experimental task, participants needed to notice the stimulus feature or regularity involved in the timing of pressing the key. Emotions induced by the experience of noticing related to goals and rewards may have gradually enhanced the SoA. However, it remains unclear whether noticing the stimulus regularity induces rewards and may affect SoA. The purpose of this study was to investigate the influence over time of the experience of noticing during a perceptual-motor task on the SoA. To investigate this influence, we modified our previous experiment. Participants were instructed to stop a moving object by a keypress when it reached the center of a target on the screen. The moving speed changed with an implicit regularity. Participants performed the perceptual-motor task and an IB task simultaneously. After completing the experiment, participants answered questions about whether they noticed the implicit regularity. Using this procedure, it was possible to separate participants between a group noticing the stimulus regularity and a non-noticing group. We expected the IB effect to gradually enhance in the noticing group compared to that in the non-noticing group.

## 2. Materials and Methods

### 2.1. Participants

The sample in this study was 29 right-handed, healthy participants (14 females, mean age = 21.2 ± 0.7). All the participants reported the vision, hearing, verbal, and finger functions required for the experiment. The Kio University Ethics Committee approved the study’s procedures (approval number: H28-50), and researchers conducted the experiment in accordance with the Declaration of Helsinki. All participants gave written informed consent before participating in the experiment.

### 2.2. Materials

LabVIEW (National Instruments, Japan) was used to create the IB paradigm. A personal computer (Twotop original PC, Unitcom, Japan) was used for all tasks and to record the data. A 23-inch display (1098 × 630 px) was used as the screen (Mitsubishi Electric, Tokyo, Japan), the size of the stimulus’s visual angle (deg) was 43.6° horizontally and 21.8° vertically, and the viewing distance was 75 cm.

### 2.3. Procedure

#### 2.3.1. Practice Task

First, the participants completed a practice exercise to become familiar with the experimental task’s set-up. The time interval between a keypress with the right index finger and a tone sounding (50 ms, 900 Hz) was 1–1000 ms. In ms units, participants verbally estimated the time interval between the keypress and the sound. This practice task was composed of 24 trials. Time intervals were random for each trial and, after responding, each participant received feedback on the actual interval. This task was excluded from subsequent analyses because it was just for practice.

#### 2.3.2. Time Baseline Task

To control individual differences in temporal perception, a time baseline task was established based on the designs used in the previous literature [25,26,27]. The appropriate number of trials required for this study was determined by referring to the literature [25,28,29]. The participants sat in front of a monitor and performed 18 trials where they had to estimate the time interval between two sounds composed of 200, 500, or 700 ms, which was chosen at random for each trial. In ms units, participants verbally estimated the time interval between the two sounds. The mean value obtained from 18 trials was used as the baseline. The participants did not know the actual time interval.

#### 2.3.3. Experimental Motor Task

**Figure 1:**
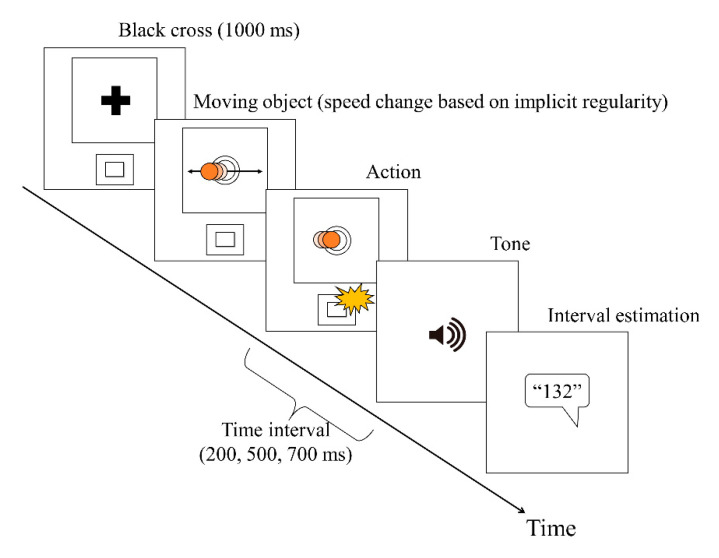
Experimental motor task.

We arranged the previous study [25] of an IB task with implicit regularity in a perceptual-motor task. First, a black fixation cross was presented on the screen for 1 s. Next, a red, circular, flat object repeatedly moved horizontally across the screen. The participants pressed a key when the object reached the center of the screen. The object had a radius of 20 px, and the circle in the center of the screen had a radius of 30 px. The target stopped moving as soon as the participant pressed the key, and after a time interval, a sound was presented. The participant estimated the time interval between pressing the key and hearing the sound. As in the time baseline task, a time interval of 200, 500, or 700 ms, respectively, was chosen randomly for each trial, and participants verbally estimated the time interval between pressing the key and hearing the sound. This task comprised of 10 blocks (18 trials per block). For participants noticing the implicit regularity of the perceptual-motor task, we changed the moving speed of the object. The moving speed (visual angle) was in five stages (Speed 1, 7.09 degrees/s; Speed 2, 14.13 degrees/s; Speed 3, 21.06 degrees/s; Speed 4, 27.84 degrees/s; and Speed 5, 34.43 degrees/s). The speed was gradually changed from Speed 1 to Speed 5 every 1 s. After Speed 5, the speed was set to Speed 1 again. This loop was repeated until the participants pressed a key. As a result of the preliminary experiments, by setting the regularity, we were able to divide the participants into two groups: the noticing group, who noticed the regularity, and the non-noticing group, who did not notice the regularity. Notably, the participants had no knowledge of the regularity before the experiment. After completing all trials, the participants answered questions regarding whether and when they noticed the implicit regularity. On the basis of the answers, we clearly divided the participants into two groups: the noticing group, who noticed the regularity, and the non-noticing group, who did not notice the regularity (see Figure 1).

### 2.4. Data Analysis

The distance of error (px) between the object’s center and the target’s center was calculated, with lower error values indicating higher motor performance; the mean value for each block represented the error. By using the first block as the baseline, the ratio with each block was calculated as the adaptation effect. High values indicate high adaptation. An assumption was that the adaptation effect was higher in the noticing group than in the non-noticing group.

The temporal perception varies depending on the participant. Thus, to eliminate bias associated with individual differences in temporal perception, the mean value of each block from the time baseline task was subtracted from the IB value for each block from the experimental task: (estimated time interval in the experimental task − actual time interval in the experimental task) – (estimated time interval in the time baseline task − actual time interval in the time baseline task). Low IB values indicate enhancement of SoA. The calculation followed the literature [25]. The mean value for each block (all 18 trials) was calculated.

The nine blocks were divided into the early phase (blocks 2–4), middle phase (blocks 5–7), and last phase (blocks 8–10). The adaptation effect was analyzed using two-way mixed analysis of variance (ANOVA), accounting for the group (noticing, non-noticing), phase (early, middle, last), and interaction. The Bonferroni method was used for post hoc comparisons.

Similarly, to investigate the time series change of IB, the nine blocks were divided into the early phase (blocks 2–4), middle phase (blocks 5–7), and last phase (blocks 8–10). IB was analyzed using a two-way mixed ANOVA that accounted for the group (noticing, non-noticing), phase (early, middle, last), and interaction. The Bonferroni method was used for post hoc comparisons. *p* values < 0.05 were considered statistically significant.

Additionally, the relationship between the adaptation effect and IB in each phase was analyzed using Pearson’s correlation coefficient in all participants and within each group. *p* values < 0.017 were considered statistically significant, according to the Bonferroni correction. SPSS Statistics for Windows ver. 24 (IBM, Tokyo, Japan) was used as the analysis software.

## 3. Results

**Figure 2:**
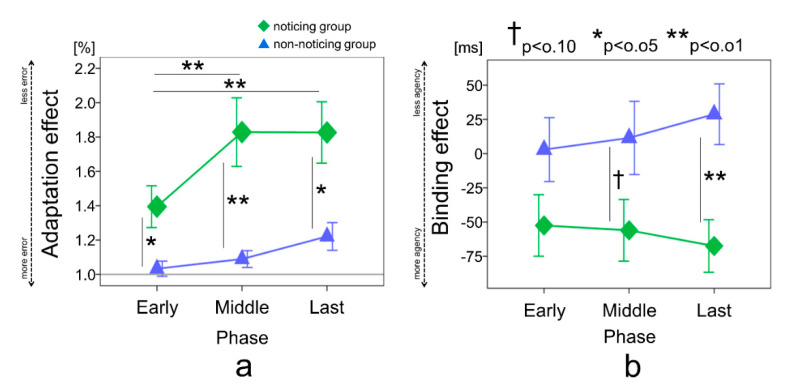
(**a**) Adaptation effect for each group in each phase. 1.0 indicates the baseline. (**b**) Binding effect for each group in each phase. The data represent the means ± standard error; † indicates a *p*-value < 0.1; * indicates a *p*-value < 0.05; ** indicates a *p*-value < 0.01; green square plot = noticing group; blue triangle plot = non-noticing group.

The participants were divided into a noticing group (*n* = 17) and a non-noticing group (*n* = 12). Most participants in the noticing group noticed the regularity in the early phase. For the adaptation effect, the results of a two-way mixed ANOVA showed significant main effects by the group factor (F(1, 27) = 8.70; *p* = 0.007, effect size (η_p_^2^) = 0.24), the phase factor (F(2, 54) = 10.69; *p* < 0.001, effect size (η_p_^2^) = 0.28), and the interaction (F(2, 54) = 3.67; *p* = 0.03, effect size (η_p_^2^) = 0.12). In the comparison between groups, the adaptation effect was higher in the noticing group than in the non-noticing group in the early phase (mean difference = 0.36, 95% CI = 0.05 to 0.67, *p* = 0.02), middle phase (mean difference = 0.73, 95% CI = 0.24 to 1.23, *p* = 0.005), and last phase (mean difference = 0.60, 95% CI = 0.14 to 1.06, *p* = 0.01). In the comparison between the phases of the noticing group, the adaptation effect in the middle phase (mean difference = 0.43, 95% CI = 0.18 to 0.68, *p* < 0.001) and last phase (mean difference = 0.43, 95% CI = 0.19 to 0.67, *p* < 0.001) was significantly higher than in the early phase, while no significant difference was observed in the middle or last phases (mean difference = 0.02, 95% CI = −0.19 to 0.20, *p* = 1.00). In a comparison between the phases of the non-noticing group, no significant differences were observed in the early or middle phases (mean difference = −0.05, 95% CI = −0.35 to −0.24, *p* = 1.00), the early and last phases (mean difference = −0.18, 95% CI = −0.47 to −0.09, *p* = 0.31), or the middle and last phases (mean difference = −0.13, 95% CI = −0.37 to 0.10, *p* = 0.51) (Figure 2a).

For the binding effect, the results of a two-way mixed ANOVA showed a significant main effect for the group factor (F(1, 27) = 5.48; *p* = 0.02, effect size (η_p_^2^) = 0.16) and the interaction (F(2, 54) = 3.18; *p* = 0.04, effect size (η_p_^2^) = 0.10). No significant difference was observed for the phase factor (F(2, 54) = 0.21; *p* = 0.80, effect size (η_p_^2^) = 0.008). In the comparison between groups, no significant difference was observed in the early phase (mean difference = −55.42, 95% CI = −123.54 to 12.69, *p* = 0.10). However, a close-to-significant difference was observed in the middle phase (mean difference = −67.50, 95% CI = −139.27 to 4.26, *p* = 0.06), and a significant difference was observed in the last phase (mean difference = −97.27, 95% CI = −156.84 to −35.70, *p* = 0.003) (Figure 2b).

In the correlation analysis between the adaptation effect and IB in all participants, there were no significant correlations in the early phase (*r* = −0.18, *p* = 0.34) or middle phase (*r* = −0.25, *p* = 0.17). Instead, there was a close-to-significant negative correlation in the last phase (*r* = −0.41, *p* = 0.028). In the correlation analysis in the noticing group, there was no significant correlation in the early phase (*r* = −0.09, *p* = 0.72), middle phase (*r* = −0.15, *p* = 0.55), or last phase (*r* = −0.21, *p* = 0.40). In the correlation analysis in the non-noticing group, there was no significant correlation in the early phase (*r* = 0.07, *p* = 0.82), middle phase (*r* = 0.16, *p* = 0.61), or last phase (*r* = −0.30, *p* = 0.33).

## 4. Discussion

We were able to divide the participants into noticing and non-noticing groups using this task. Therefore, we were able to adjust the conditions to investigate the influence of the experience of noticing regarding the perceptual-motor task on SoA. The result of the high adaptation effect in the noticing group indicates that the participants noticed the regularity. In addition, the result of the difference in the IB effect between the groups supported our hypothesis: the noticing group showed an enhancement of SoA. Notably, SoA was gradually enhanced in the noticing group, whereas SoA gradually decreased in the non-noticing group. Although many previous studies reported that stimulus regularity affected performance and response time [30,31], this study has a novel finding; that is, stimulus regularity influenced SoA, which is an aspect of embodiment.

Kelso argued that the eureka experience of the transition, when agents realize that their actions are coupled with changes in the environment, may be the origin of SoA [16]. In this study, while repeating the trial in the noticing group, there seemed to be a transition to when participants noticed as a result of the coupling of actions and outcomes of the motor task. This transition may cause a eureka-like effect and contribute to the enhancement of SoA in the noticing group. By contrast, because the non-noticing group had no such transition, SoA may have decreased in this group.

The result indicated that the IB effect was enhanced with adaptation, which is consistent with our previous findings [25]. It is suggested that SoA may arise in a process with reduced error. The most notable findings are that (1) because participants noticed the regularity, SoA was gradually enhanced, and (2) because participants did not notice the regularity, SoA gradually decreased. The results of our previous study, in which participants with a high adaptation effect had high SoA with perceptual-motor adaptation, are comparable with (1). Notably, (2) represents novel data that demonstrates the temporal process of decreasing SoA. From the results in (2), we propose a new interpretation: SoA gradually decreased as mismatching of the motor task continued. Few studies have examined the temporal decrease (or enhancement) of SoA. Di Costa et al. investigated the SoA of rewards during the performance of an adaptation task and observed that the SoA involved an enhanced reward system [32]. Based on the above discussions, positive emotion driven by the reward system may have enhanced the SoA. Meanwhile, motivation may have decreased gradually when there was no noticing. As a result, the SoA may also have gradually decreased.

In the correlation analysis, in all participants, the close-to-significant negative correlation in the last phase might partly support the relationship between SoA and motor control. This result suggests that SoA may be involved in the ability to correct errors. The absence of correlations in the early and middle phases suggests that the relationship between SoA and motor control may change over time. In contrast, the reason why there was no correlation in the within-group analysis may be because the grouping reduced the variance. These interpretations are speculative, and further experimental approaches are needed to investigate the relationship between SoA and motor control. Although this experimental approach had to use the average value for each block in the analysis to ensure validity, a time series analysis for each trial would be needed in future research.

Two possible objections should be considered. The first is that the participants did not have knowledge of the regularity. The participants answered when noticing the regularity after completing all the tasks. A limitation of our study is that we could not measure the moment of noticing. If we could measure the behavior data of the moment of noticing, we could pursue the relationship between awareness and SoA in detail. Furthermore, to investigate the hypothesis that noticing changes the SoA, verification is needed of whether the SoA would be enhanced by teaching the regularity to the non-noticing group. In further research, we plan to investigate whether there is a difference in change of the SoA in cases where the participants notice the regularity by themselves or through teaching by others. The second is that it is unclear how SoA interacts with motor performance and changes over time. We have reported in a case series that SoA is related to the recovery processes of motor paralysis after a stroke [33]. Thus, it is necessary to discuss the relationship between SoA and the processes of functional recovery after a brain injury. This discussion might explain the changes in SoA over time.

## 5. Conclusions

In this study, we successfully developed a novel task to measure IB and motor performance, including the noticing element, discovered that it was possible to divide the participants into noticing and non-noticing groups using this task, and suggested that the experience of noticing the stimulus feature of the motor task may enhance SoA.
